# Cheminformatics-Based Discovery of Potential Chemical Probe Inhibitors of Omicron Spike Protein

**DOI:** 10.3390/ijms231810315

**Published:** 2022-09-07

**Authors:** Salman Ali Khan, Alamgir Khan, Komal Zia, Ihab Shawish, Assem Barakat, Zaheer Ul-Haq

**Affiliations:** 1 Dr. Panjwani Center for Molecular Medicine and Drug Research, International Center for Chemical and Biological Sciences, University of Karachi, Karachi 75270, Pakistan; 2 H.E.J. Research Institute of Chemistry, International Center for Chemical and Biological Sciences, University of Karachi, Karachi 75270, Pakistan; 3 Department of Math and Sciences, College of Humanities and Sciences, Prince Sultan University, P.O. Box 66833, Riyadh 11586, Saudi Arabia; 4 Department of Chemistry, College of Science, King Saud University, P.O. Box 2455, Riyadh 11451, Saudi Arabia

**Keywords:** Omicron, virtual screening, MD simulation, DFT, COVID-19, SARS-CoV-2 variants

## Abstract

During the past two decades, the world has witnessed the emergence of various SARS-CoV-2 variants with distinct mutational profiles influencing the global health, economy, and clinical aspects of the COVID-19 pandemic. These variants or mutants have raised major concerns regarding the protection provided by neutralizing monoclonal antibodies and vaccination, rates of virus transmission, and/or the risk of reinfection. The newly emerged Omicron, a genetically distinct lineage of SARS-CoV-2, continues its spread in the face of rising vaccine-induced immunity while maintaining its replication fitness. Efforts have been made to improve the therapeutic interventions and the FDA has issued Emergency Use Authorization for a few monoclonal antibodies and drug treatments for COVID-19. However, the current situation of rapidly spreading Omicron and its lineages demands the need for effective therapeutic interventions to reduce the COVID-19 pandemic. Several experimental studies have indicated that the FDA-approved monoclonal antibodies are less effective than antiviral drugs against the Omicron variant. Thus, in this study, we aim to identify antiviral compounds against the Spike protein of Omicron, which binds to the human angiotensin-converting enzyme 2 (ACE2) receptor and facilitates virus invasion. Initially, docking-based virtual screening of the in-house database was performed to extract the potential hit compounds against the Spike protein. The obtained hits were optimized by DFT calculations to determine the electronic properties and molecular reactivity of the compounds. Further, MD simulation studies were carried out to evaluate the dynamics of protein–ligand interactions at an atomistic level in a time-dependent manner. Collectively, five compounds (AKS-01, AKS-02, AKS-03, AKS-04, and AKS-05) with diverse scaffolds were identified as potential hits against the Spike protein of Omicron. Our study paves the way for further in vitro and in vivo studies.

## 1. Introduction

The COVID-19 pandemic, due to SARS-CoV-2 virus, has socially and economically affected the world, representing the greatest threat to human health and the economy in more than a century [1,2]. Considering the potential risk and contagiousness of the SARS-CoV-2, lockdowns and other preventive measures including, social distancing, surface disinfectant, hand hygiene, and personal protective equipment have been implemented all around the world to reduce the spread of this deadly virus [3,4,5,6,7,8,9,10]. Despite such preventive majors, variants of this deadly virus, including, Alpha, Beta, Gamma, Delta, Kappa, and others, have emerged repeatedly and has spread worldwide [11,12,13]. These variants have influenced the periodic waves of infection that occur asynchronously in various regions of the world [14,15].

At the end of 2021, while the Delta wave was subsiding, the new and genetically distinct lineage of SARS-CoV-2 emerged in Botswana and South Africa [16]. In November 2021, the newly emerged SARS-CoV-2 variant from both South Africa and Botswana was designated as belonging to a new PANGO lineage (B.1.1.529), which was later divided into sublineages; BA.1 (the main clade), BA.2 and BA.3 [17]. On 26 November 2021, the lineage was named Omicron and designated as the fifth variant of concern by the WHO on the recommendation of the Technical Advisory Group on SARS-CoV-2 Virus Evolution. The emergence of Omicron has raised major concerns regarding increased infectivity, immune escape capabilities, and the possibility of reinfection [18,19,20]. Many countries have also implemented travel restrictions to prevent the rapid spread of the Omicron.

The emergence of Omicron has rapidly gained the attention of academic research and scientists around the world owing to more than thirty mutations in Spike glycoprotein with 15 mutations in the receptor-binding domain, attachment factor for human ACE2 receptor and many viral neutralizing antibodies [21]. The Spike glycoprotein consists of two S1 and S2 subunits. S1 contains an N-terminal domain (NTD), a signal peptide (SP) at the N terminus, and a receptor-binding domain (RBD), and serves the function of receptor binding. S2 contains a fusion peptide (FP) domain, internal fusion peptide (IFP), two heptad-repeat domains (HR1 and HR2), a transmembrane domain, and a C-terminal domain, and functions in membrane fusion to facilitate cell entry [22]. The Spike protein undergoes two fundamental cleavage events, the first splitting S1 and S2 and the second splitting S2 into FP and S2′. Cleavage of Spike protein into S1 and S2 is the prerequisite in virus entrance into a host cell, which must take place prior to viral fusion with the host cell membrane [23]. The receptor-binding domain contains a receptor-binding motif (RBM), region of a few amino acid residues that interacts directly with the human ACE2 receptor on the surface of human cells, mediating virus invasion and determining virus transmissibility [24,25]. Furthermore, convalescent plasma, vaccinations, and monoclonal antibodies (mAbs) all target the Spike protein for neutralization [26]. Thus, Spike protein, specifically the S1 subunit, is a primary target for vaccine development and antibody-based therapeutics against all the SARS-CoV-2 variants.

In a recent study, the ACE2-targeting antibody has been demonstrated to reduce the Omicron variant at the cellular level, suggesting that this is a promising avenue of investigation [27]. Recently, an important RBD-targeted biparatopic nanobody demonstrated improved neutralizing efficacy against Omicron and other variants [28]. Furthermore, modified extracellular vesicles (EVs) fused with palmitoylated ACE2 (PM-ACE2-EV) demonstrated the neutralization potency against authentic SARS-CoV-2 both in vitro and in vivo [29]. Various antiviral drugs and possible potential treatments using experimental and theoretical techniques are also being examined against Omicron and other SARS-CoV-2 variants [30,31,32,33,34]. Takashita and co-authors investigated the reactivity of three antiviral drugs (Remdesivir, Molnupiravir, and PF-07304814) and the seven monoclonal antibodies by means of enzyme-linked immunosorbent assay (ELISA) coated with recombinant Spike protein derived from the Wuhan strain, and other variants including, Alpha, Beta, Gamma, Delta, and Omicron variants. The results suggested that all the monoclonal antibodies that were tested, neutralized the Wuhan strain, Alpha and Delta variants while all three antiviral drugs showed efficacy against all the variants including the Omicron variant [35]. The mechanism of action for these drugs is different from each other which indicated that the drug sensitivity profile of the SARS-CoV-2 variants is not easily changed in response to antiviral drugs. Similarly, EIDD-1931, Ribavirin, Favipravir, Nafamostat, Camostat, and Aprotinin have been investigated against Omicron [36,37,38]. These studies provided the rationale for the design and development of new antiviral compounds to treat SARS-CoV-2 variants. Thus, in this study we employed multistep virtual screening of an in-house database of synthetic and natural compounds in combination with DFT and MD simulation to identify potential inhibitors of Spike protein of Omicron. In the result of the multistep screening strategy, five compounds were identified as potential inhibitors of Spike-ACE2 protein–protein interactions.

## 2. Results and Discussion

### 2.1. Docking-Based Virtual Screening

For docking-based screening, the X-ray crystal structure of RBD of Omicron’s Spike glycoprotein in complex with human ACE2 receptor was utilized. Analysis of the structure reveals that the RDM interacts with the hACE2 [39]. Ser19 of hACE2 forms hydrogen bonds with the Ala475 and Asn477 of RBD. Similarly, Gln24 of hACE2 mediates a hydrogen bond with Asn487 of RBD. Tyr83 of hACE2 interacts with Tyr489 and Asn287 of RBD through hydrogen bond contacts and it also mediates a π-π stacking interaction with Phe486 of RBD. Likewise, His34 of hACE2 forms hydrogen bond contact with Tyr453 of RBD. Asp38 and Gln42 of hACE2 established hydrogen bonds with Tyr449 of RBD and Asp38 was also found to mediate a salt bridge with Arg498 of RBD. Tyr41 and Lys353 of hACE2 form hydrogen bonds with Trp500 and Gly502 of RBD, respectively. Similarly, Tyr41 also mediates π-π stacking interaction with Tyr501 of RBD. Phe486 from RBD mediates hydrophobic interactions with a small hydrophobic pocket in the interface formed by Phe28, Leu79, Met82, and Tyr83 of hACE2 [40].

Defining the aforementioned interfacial residues of RBD of Omicron’s Spike protein as a docking grid, molecular docking was performed for all the prepared compounds of the in-house database. Results of the docking simulation were analyzed based on the ability of the docked compounds to inhibit the RBD-ACE2 protein-protein interaction in the best ranking binding pose. The analysis of the docking results revealed that most of the compounds are predicted to interact with interfacial residues of RBD; however, the top ranked compounds were analyzed. A total of 55 compounds were obtained based on the best ranking binding modes. The obtained compounds were further shortlisted based on the visual analysis of the interaction patterns. As a result of successive screening, the five best compounds with the highest docking scores, significant interactions and structural diversity were selected for further analysis. The 2D structures of selected hits, docking scores, and detail of interactions between protein and the selected hits are illustrated in (Table 1).

### 2.2. Analysis of Binding Modes

In the effort to design small molecule drug against SARS-CoV-2 and its spreading variants, various potential chemical compounds have been reported, having scaffold including; oxazole-carboxamides, benzamides, anthraquinone, piperidine, alkylamines, triazole, benzimidazole, carbasugars, Methylene Blue derivatives, phenolic derivatives), flavanone, and others [41,42,43,44]. The five selected virtual hits: AKS-01 (unpublished), AKS-02 [45], AKS-03 (unpublished) AKS-04 (unpublished), and AKS-05 [46], belong to the chemical class benzamide, benzothiazine, triazole, benzimidazole, and thiazol, respectively, and were previously synthesized by a synthetic group from our institute.

**Figure 1:**
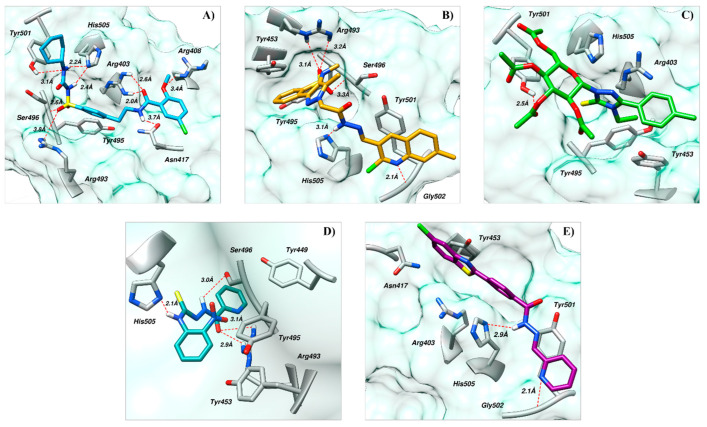
Docked poses of selected five compounds in the binding site of RBD. (**A**) AKS-01, (**B**) AKS-02, (**C**) AKS-03, (**D**) AKS-04, and (**E**) AKS-05.

AKS-01, a derivative of benzamide, in the binding site of Spike protein demonstrated nine hydrogen bond contacts with the interfacial residues of RBD with the binding affinity of −6.94 kcal/mol (Figure 1A). Oxygen and nitrogen atoms in the compound established the hydrogen bonds with Arg403, Arg408, Asn417, Ser486, Tyr501, and His505. Moreover, hydrophobic interactions were observed with the Asn417, Tyr453, and Tyr495. In addition, π-cation interaction was also observed with the Arg403. The potency of this compound may be attributed by a previous study that reported the benzamide derivative as an inhibitor of RBD-ACE2 interface with the IC_50_ of 0.7 μM [41].

Similarly, AKS-02, a derivative of benzothiazine, demonstrated the five hydrogen bonds in the binding site of Spike protein with the binding affinity of −6.46 kcal/mol (Figure 1B). Oxygen and nitrogen atoms establish hydrogen bonds with Arg493, Ser496, Gly502, and His505. Hydrophobic interactions were observed with Tyr453, Tyr495, and Tyr501. Additionally, π-stacking was observed with Tyr501. To the best of our knowledge, benzothiazine scaffold is not reported or tested yet against SARS-CoV-2.

Similarly, in the binding site of Spike protein, AKS-03, a triazole derivative, resides well by mediating one hydrogen bond and significant hydrophobic interactions with the binding affinity of −6.03 kcal/mol (Figure 1C). The oxygen atom in the compound mediates the hydrogen bond with Tyr501. Furthermore, hydrophobic interactions were observed with Tyr453, Tyr495, Tyr501, and His505. Additionally, π-cation interaction and a salt bridge were also observed with Arg403 and Arg493, respectively. Recently, Alzahrani and co-workers reported the anti-SARS-CoV-2 activity of chemical compounds having triazole moiety, which may pronounce the potency of this scaffold against COVID-19 [44].

In the case of AKS-04, a benzimidazole derivative, four hydrogen bonds were observed with the interfacial residues of RBD with an affinity of −5.47 kcal/mol (Figure 1D). Similarly, oxygen and nitrogen atoms in the compound mediate hydrogen bonds with Arg493, Ser496, and His505. Similarly, Tyr449, Tyr450, and Tyr495 mediate hydrophobic interactions to accommodate the compound firmly. π-cation interaction was also observed with Arg403. Recently, Park and Eun reported the in vitro studies of some chemical compounds having a benzimidazole scaffold using the binding bioassay between Spike protein and human ACE2 receptor [43]. The study suggested that benzimidazole is an attractive scaffold for further development as a SARS-CoV-2 entry inhibitor.

The binding pose of AKS-05, a thiazol derivative, demonstrated two hydrogen bonds and significant hydrophobic interaction with the RBD residues with the affinity of −5.24 kcal/mol (Figure 1E). The nitrogen atoms in the compound mediate hydrogen bonds with Tyr501 and His505. Moreover, hydrophobic interactions were observed with Asn417, Tyr453, Leu455, and Tyr501. In addition, two π-cation interactions were observed with Arg403 and His505. Recently, Lin and co-workers conducted research on Ceftazidime to determine its potency against COVID-19 [44]. They suggested that the unique moieties in Ceftazidime, including 2-aminothiazole might be involved in mediating the binding to S-RBD and eventually blocked the protein interaction between S-RBD and ACE2.

### 2.3. DFT Studies

The selected compounds were optimized by DFT calculations using Gaussian16 to evaluate the electronic and molecular properties [47]. The calculations were executed with B3LYP functional and 6-31G (d, p) basis set and Gaussview 5.0 was used to visualize the data.

#### 2.3.1. Molecular Orbital Analysis

**Figure 2:**
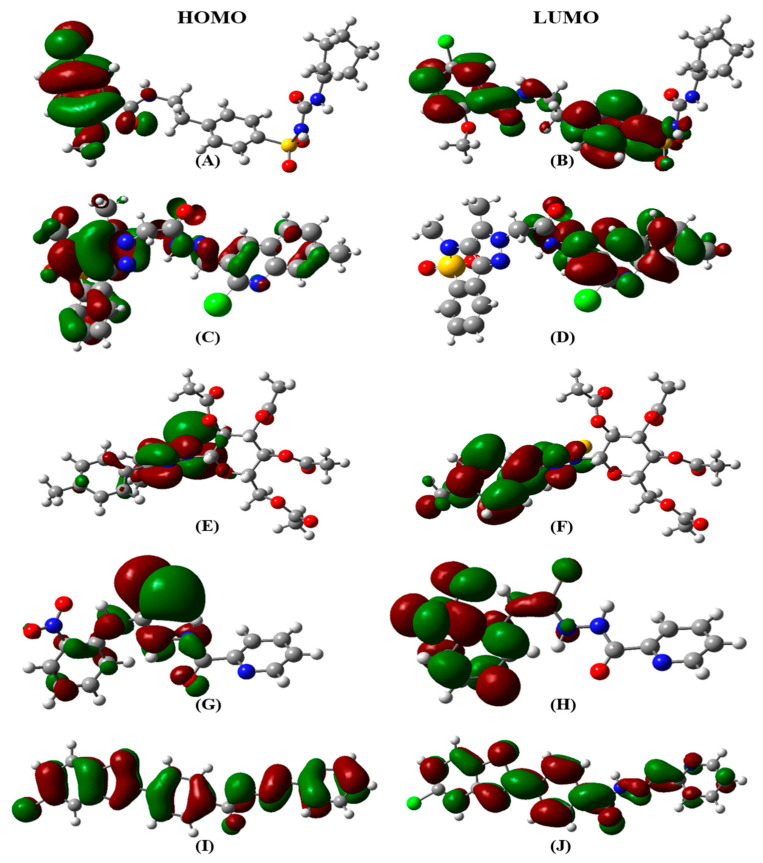
Spatial distribution of molecular orbitals for (**A**,**B**) AKS-01, (**C**,**D)** AKS-02, (**E**,**F**) AKS-03, (**G**,**H**) AKS-04, and (**I**,**J**) AKS-05.

**Figure 3:**
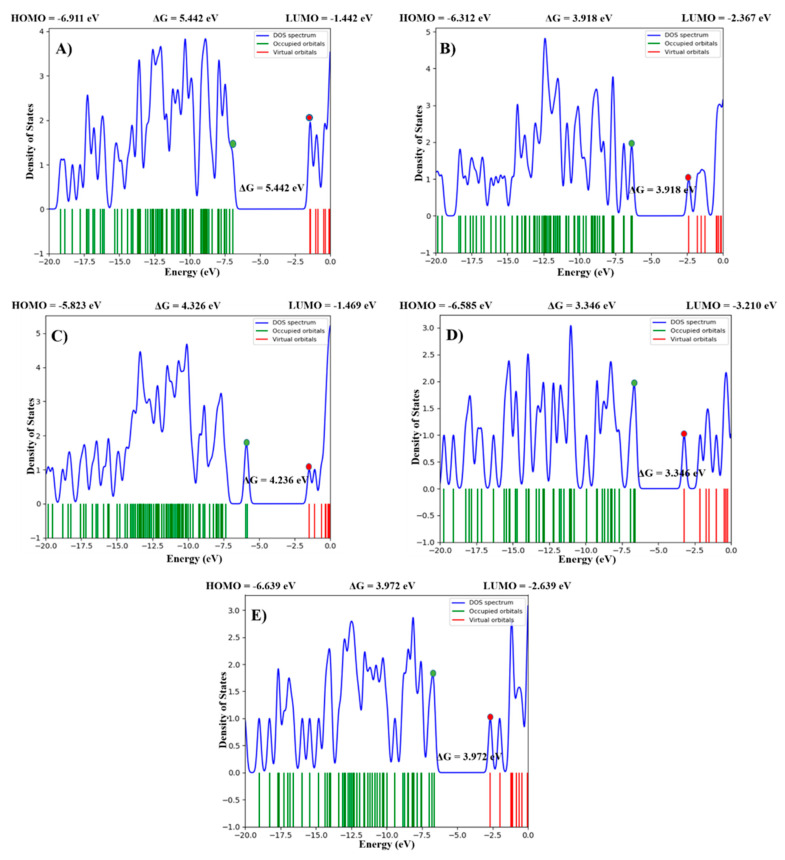
Density of states (DOS) plot of HOMO LUMO and energy gap of the five selected compounds (**A**) AKS-01, (**B**) AKS-02, (**C**) AKS-03, (**D**) AKS-04, and (**E**) AKS-05. Green and Red dots shows HOMO and LUMO orbitals.

The highest occupied molecular orbital (HOMO) and lowest unoccupied molecular orbital (LUMO) play an important role in the investigation of molecular reactivity and stability of the compounds. The difference between ELUMO and EHOMO is known as the band energy gap which depicts the stability and the intramolecular charge transfer of molecules. The lower value of band energy gap indicates the higher reactivity. (Table 2) illustrates the ELUMO, EHOMO, and their energy gap along with the dipole moment of the five selected compounds. The results indicated that among the five optimized compounds, AKS-04 possessed the highest molecular reactivity as demonstrated by the lowest band energy gap of 3.346 eV as compared to other compounds. Similarly, AKS-01 possessed the lowest molecular reactivity owing to the highest band energy gap value. AKS-02, AKS-03, and AKS-05 showed a moderate band energy gap with the value of 3.918, 4.326, and 3.972 eV, respectively. The spatial distribution of molecular orbitals for all the compounds is depicted in Figure 2 while Figure 3 depicts the density of states of HOMO and LUMO with band energy gaps for clear visualization. Moreover, AKS-03 showed the highest value of dipole moment while AKS-02 showed the lowest dipole moment. The DFT calculations also revealed that AKS-02 showed the highest optimized energy followed by AKS-01, AKS-03, AKS-05, and AKS-04.

#### 2.3.2. Molecular Electrostatic Potential Map

**Figure 4:**
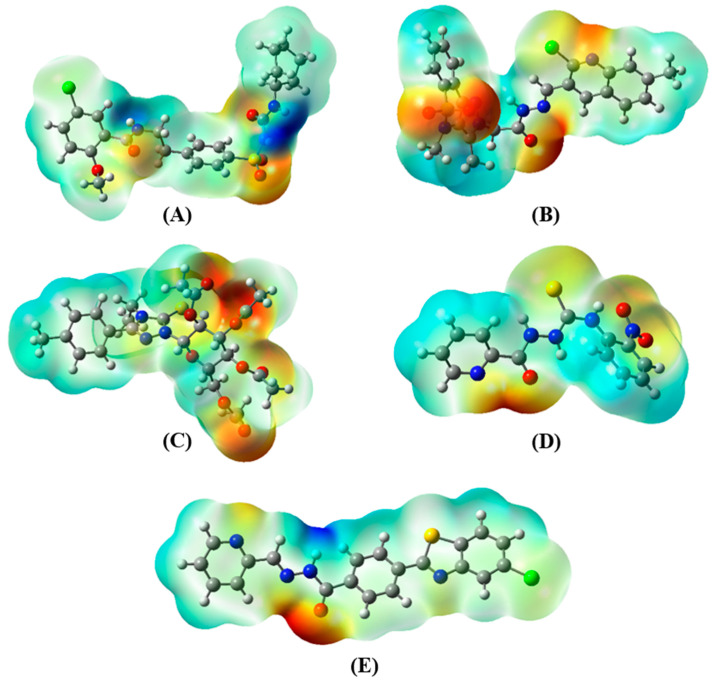
Electrostatic potential maps for (**A**) AKS-01, (**B**) AKS-02, (**C**) AKS-03, (**D**) AKS-04, and (**E**) AKS-05.

A molecular electrostatic potential (MEP) map, in the process of molecular recognition, is a valuable visual method for analyzing the relative polarity of a molecule. The MEP map of five selected compounds are presented in (Figure 4). Different colors on the map show different electrostatic potential regions. Reddish yellow color represents the electron rich or negatively charged regions while blue color represents the electron deficient or positively charged regions. Cyan to white color represents the zero potential or neutral regions. It was observed that the negative charges were located on the oxygen atoms and positive charges were located on the hydrogen atoms attached to nitrogen atoms. These negatively and positively charged regions were involved in the non-covalent interactions between the selected compounds and the Spike protein.

### 2.4. MD Simulation

The results of molecular docking studies revealed that the hit compounds interacted significantly with the crucial residues of RBM and exhibited complementarity, which indicated that the selected compounds were instrumental for the experimental identification of potent inhibitors of Spike protein of Omicron. Nevertheless, molecular docking studies do not give information on the time-dependent stability of interactions, the effect of solvent and intrinsic dynamics of the protein. Therefore, MD simulations were executed for five selected compounds in complex with RBD to probe the stability of protein–ligand interactions. The analysis was attained by examining the Root Mean Square Deviation (RMSD), Root Mean Square Fluctuation (RMSF), Radius of Gyration (RoG), and hydrogen bond interactions.

**Figure 5:**
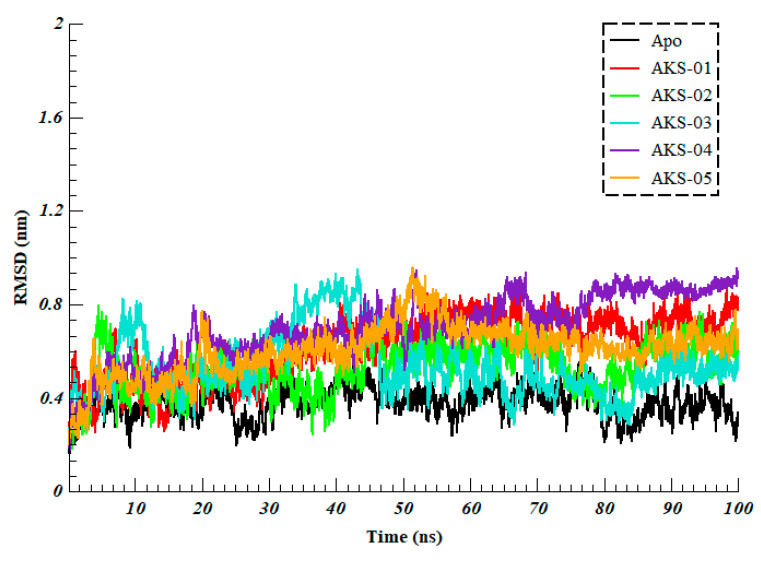
Root Mean Square Deviation of backbone carbon atoms of Apo (Omicron’s RBD) and five selected compounds in complex with RBD over the 100 ns of simulation time.

To evaluate the stability of protein–ligand complexes, RMSD of the backbone carbon atoms was calculated. It was evident from (Figure 5), all the complexes experience different degrees of fluctuations. All the systems fluctuated between 0.30 to 0.80 nm and converged after 50 ns with inconsiderable fluctuations until the end of the simulation. Average RMSD was calculated after the convergence and it was found that the Apo system projected the most stable RMSD with the average value of 0.37 ± 0.06 nm while AKS-01, AKS-02, AKS-03, AKS-04, and AKS-05 in complex with RBD projected the average RMSD of 0.72 ± 0.05 nm, 0.57 ± 0.07 nm, 0.48 ± 0.07 nm, 0.80 ± 0.07 nm and 0.66 ± 0.07 nm, respectively. These results indicated that the selected compounds interact significantly with the RDB residues, crucial for the attachment of hACE2 and stabilized the target protein.

**Figure 6:**
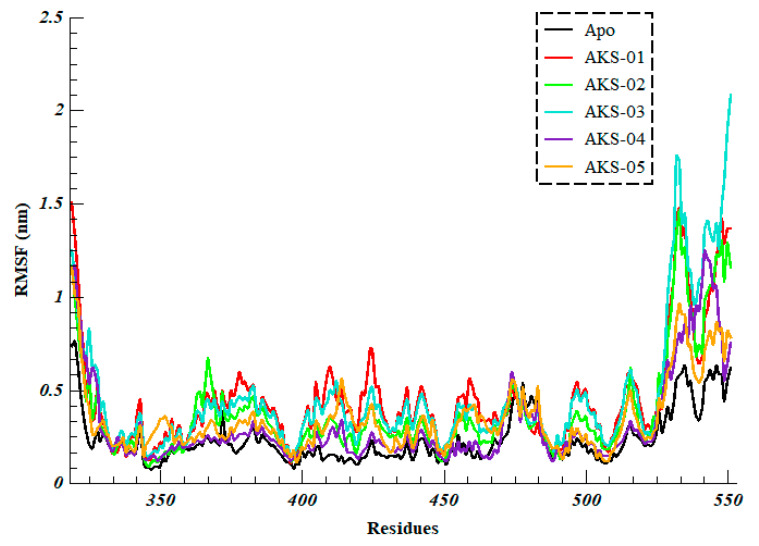
Root Mean Square Fluctuation of residues of Apo (Omicron’s RBD) and five selected compounds in complex with RBD over the 100 ns of simulation time.

To further evaluate the intrinsic and ligand-induced flexibility/rigidity of RBD residues, Root Mean Square Fluctuation was calculated over the 100 ns of simulation. It was observed that all the systems showed a similar pattern of fluctuations; however, the magnitude was different (Figure 6). The Apo system projected the average RMSF of 0.23 ± 0.13 nm with the least fluctuations while AKS-01, AKS-02, AKS-03, AKS-04 and AKS-05 in complex with RBD projected the average RMSF of 0.44 ± 0.28 nm, 0.37 ± 0.27 nm, 0.46 ± 0.34 nm, 0.31 ± 0.24 nm, and 0.34 ± 0.18 nm, respectively. It was interesting to note that binding site residues experience major fluctuations upon binding of the selected hit compounds in comparison to the Apo system. This observation indicated that interfacial residues of RBD fluctuate to accommodate the compounds in the binding site.

**Figure 7:**
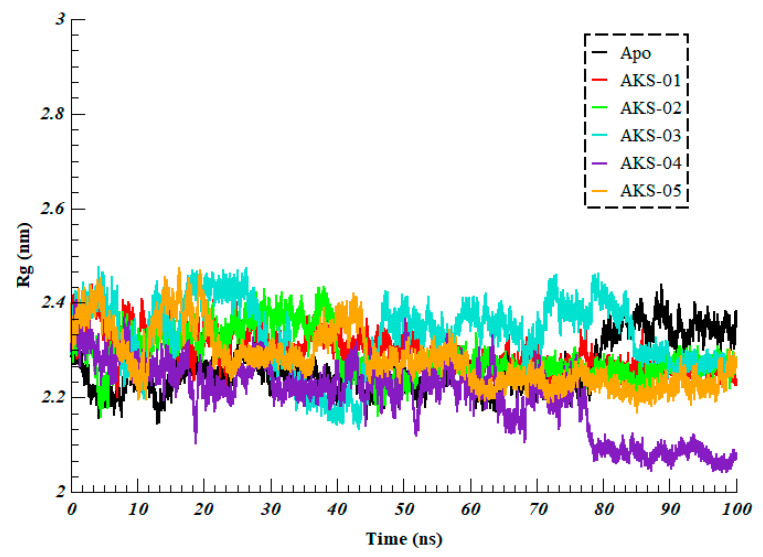
Radius of Gyration of Apo (Omicron’s RBD) and five selected compounds in complex with RBD over the 100 ns of simulation time.

The Radius of Gyration (RoG) imparts the knowledge of compactness and unfolding of protein structure upon binding of the ligands. Thus, to determine the compactness of the RDB upon binding of selected hit compounds, RoG was calculated. Higher RoG values explain less compactness (more unfolded) with high conformational entropy while low RoG values show high compactness and more stability in the structure (more folded). As evident from (Figure 7), all the simulated systems projected the RoG between 2.0 to 2.5 nm. The average RoG for Apo, AKS-01, AKS-02, AKS-02, AKS-04, and AKS-05 was found to be 2.26 ± 0.05 nm, 2.29 ± 0.04 nm, 2.29 ± 0.04, 2.33 ± 0.07 nm, 2.20 ± 0.07 nm, and 2.28 ± 0.05 nm, respectively. Initially, all the systems showed fluctuations; however, after 50 ns AKS-01, AKS-02, and AKS-05 projected the most stable RoG. The data revealed that all the systems were significantly compact throughout the simulation, which indicates that the systems are well converged.

**Figure 8:**
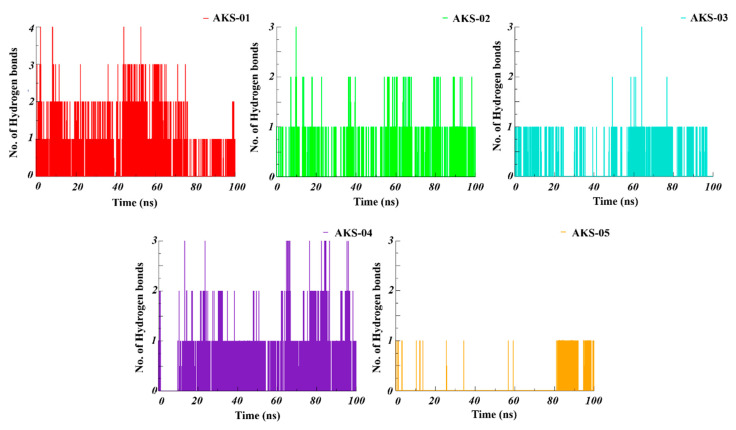
Hydrogen bonds between the interfacial residues of RBD residues and the five selected compounds throughout the 100 ns of simulation.

Inter and intra hydrogen bond interactions play a crucial role to accommodate the ligand at the active site of a protein target. To evaluate the inter-hydrogen bond interactions between the interfacial residues of RBD and five selected hit compounds, hydrogen bonds throughout the simulation time were calculated. As evident from (Figure 8), the AKS-01 mediates the highest number of hydrogen bond contacts over the simulation time. Similarly, AKS-02, AKS-03, and AKS-04 maintain three hydrogen bond contacts throughout the simulation while AKS-05 maintains only one hydrogen bond.

### 2.5. In Silico ADMET Analysis

**Figure 9:**
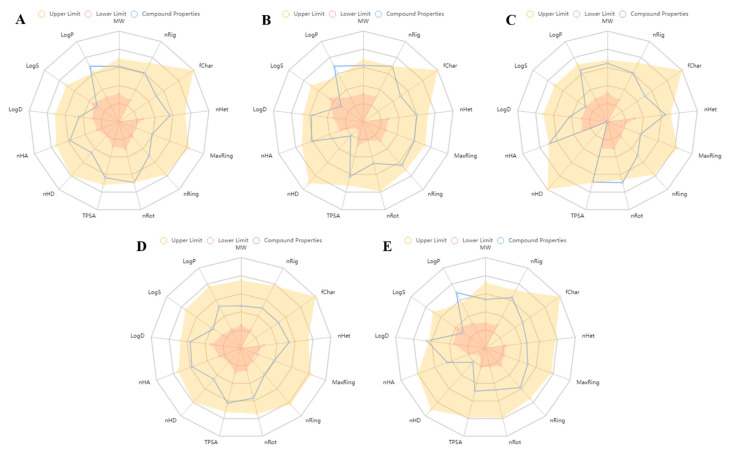
A radar plot of physiochemical properties of selected virtual hits (**A**) AKS-01, (**B**) AKS-02, (**C**) AKS-03, (**D**) AKS-04, and (**E**) AKS-05 predicted by ADMETlab.

Poor pharmacokinetics and toxicity of a drug candidate are the main reasons for the clinical failure of drug development. In the earlier process of drug development, in silico prediction of ADMET properties of lead compounds is an alternative to experimental techniques to increase the success rate of clinical development. Herein, physiochemical, pharmacokinetics, toxicity, and cross-reactivities of the five selected compounds were evaluated by ADMETlab 2.0 and the obtained results are presented in Table 3. The physiochemical properties of the compounds were evaluated by plotting a radar summarizing 13 physicochemical properties, including MW (mol. weight), nHA (number of hydrogen bond acceptor), nHD (number of hydrogen bond donor), nRot (number of rotatable bonds), nRing (number of rings), nHet (number of heteroatoms), MaxRing (number of atoms in the biggest ring), fChar (formal charger), nRig (number of rigid bonds), TPSA (topological polar surface area), LogS (log of aqueous solubility), LogP (log of the octanol/water partition coefficient), LogD (logP at physiological pH 7.4). A physiochemical range (lower and upper limit) on each axis was depicted as a pink and peach color, respectively, in which the compound properties have to fall entirely to be considered as a potential lead candidate. A radar plot of five selected virtual hits showed all the physiochemical properties in an acceptable range (Figure 9). However, LogP for AKS-01, AKS-02, and AKS-05 was out of its optimal range. Similarly, all the compounds were predicted to follow the Lipinski rule of five except AKS-03 with two violations (molecular weight and number of hydrogen bond acceptors). All the compounds were predicted to have high gastrointestinal absorption and blood–brain permeation with the exception of AKS-04. P-glycoprotein (pg) is a key member of ATP-binding cassette transporters or ABC-transporters, thus is a key to predict active efflux through biological membranes. All the compounds were found to be the substrate of pg except AKS-01. To further evaluate the toxicity of the selected compounds, the acute toxicity rule and AMES toxicity or carcinogenicity were predicted. Surprisingly, all the compounds showed 0 alerts for acute toxicity; however, AKS-04 and AKS-05 were found be to carcinogenic.

To evaluate the cross-reactivities of the selected virtual hit compounds, pan assay interface compounds, molecules that react nonspecifically with numerous biological targets rather than specifically affecting one desired target, were predicted. Surprisingly, all the compounds were predicted to be non-cross reactive and specific.

Collectively, the results of docking, MD simulation, ADMET analysis, and previous studies suggested the potencies of all five compounds. All the compounds are worth being considered for further development to optimize the inhibitory and antiviral activity. It would be interesting to optimize the benzothiazine scaffold as it is not previously reported against SARS-CoV-2.

## 3. Materials and Methods

### 3.1. Target Structure Preparation

For target structure, the RBD of Omicron in complex with human ACE2 receptor was retrieved from Protein DataBank under the accession code of 7WBP, with the resolution of 3.0 Å solved by crystallography [39]. The structure was subjected to structure preparation using the Protein Preparation wizard of Molecular Operating Environment (MOE v2019.01). Using the AMBER99 force field, this preparation entails assigning the correct bond order, removing unnecessary water molecules, terminal capping, insertion of missing atoms, energy minimization, and assigning partial charges. At the typical protonation condition (pH 7) missing hydrogen atoms were also incorporated.

### 3.2. Database Preparation

An in-house database of ~14,000 biologically active chemical compounds was utilized to extract the potential virtual hits against Omicron. The database is curated with ~2000 isolated natural small molecules and ~12,000 synthetic small molecules and peptides by researchers at our institute. The database is in SD file format along with metadata such as chemical structure, class of compound, source of compound, physical properties and biological activity, including antiviral, anti-microbial, anti-diabetic, anti-cancer, and anti-inflammatory. The compounds were converted into 3D format (mol2) from 1D format (sdf) using OPENBABEL package [48]. The converted compounds were subjected to geometry correction and protonation using MOE. Hydrogens were added and partial charges were applied, where required. Subsequently, the compounds were energy minimized using MMFF94x force field in order to correct the geometry and remove the bad clashes. Finally, the compounds were saved in mdb (MOE database) format for further processing.

### 3.3. Docking-Based Virtual Screening

Herein, a docking-based virtual screening strategy was employed to identify potential hit compounds against Omicron. A docking grid was generated by selecting the residues of RDB, present at the interface of the RBD–ACE2 complex and defined as the docking site. Afterwards, the prepared compounds of the in-house database were docked into the defined grid by the rigid protocol using Triangular Matcher as a placement method, London dG as scoring and GBVI/WSA dG as rescoring function. To evaluate the summary of protein–ligand contacts, the docked complexes were subjected to PLIF (Protein–Ligand Interactions Fingerprinting) package of MOE.

The first criteria to prioritize the compounds was based on the interaction fingerprints with the crucial residues of RDB including; Asn417, Ser446, Tyr449, Tyr453, Leu455, Phe486, Asn487, Tyr489, Arg493, Ser496, Arg498, Thr500, Tyr501, Gly502, and His505. As a result, 82 compounds were filtered out for further analysis. The second filtration criteria were based on the docking score; the top ranked compounds with the docking score in the range of −6.9 to −5.0 kcal/mol were selected which led to the extraction of 55 virtual hits. The third criteria were based on the visual analysis of the compounds using PLIP webserver and Chimera software [49]. The analysis process entailed the visual inspection of hydrogen bonding and hydrophobic interactions with the specific crucial residues and complementarity between ligand and the binding site of the Spike protein. In the result of multistep screening, five compounds were identified as potential virtual hits and further subjected to MD simulation to study dynamic behavior and protein–ligand stability.

### 3.4. Density Functional Theory

DFT calculation has recently emerged as a valuable method for investigating molecular properties and reactivity [50,51]. The orbital energies calculation offers useful information regarding the electrostatic characteristics of the compounds such as inhibitors or activators. There are considerable evidences that Density Functional Theory (DFT) offers an accurate representation of electrical and structural characteristics by calculating the electronic structure of a molecule of interest. In this study, the docked conformations of selected compounds were utilized as inputs for DFT calculations. All the calculations were performed using Gaussian16. To calculate the orbital energies, a B3LYP functional and 6-31G(d,p) basis set was used, which provided information regarding the capacity of the small molecules to transfer their energies from a HOMO (electron donor) to a LUMO (electron acceptor). These electrostatic property calculations could deliver useful data for designing new inhibitors.

### 3.5. Molecular Dynamics Simulation

All-atom MD simulations studies of the best-docked five selected compounds in complex with Omicron’s RBD were performed using GROMACS v2021.1 with the CHARMM27force field [52]. The topologies of the compounds were generated by using ACPYPE. The simulation box was defined as being 10 Å from the centrally located protein–ligand complex. To solvate the system, the box was filled with a TIP3P water model, and subsequently counter ions were added to neutralize the system. For minimization of the system, the steepest descent algorithm with maximum force < 1000 kJ mol^−1^ nm^−1^ was used. All the bonds were constrained by using the LINCS algorithm while PME algorithm was utilized to treat the long range electrostatic interactions. Equilibration was carried out in NVT and NPT ensemble at the physiological temperature 300 K, for a period of 100 ps. During the simulation, temperature and pressure were kept maintained by utilizing the Berendsen thermostat and Parrinello–Rahman pressure coupling. Finally, MD production runs were executed for 100 ns for all the well equilibrated systems. A graphical processing unit (GPU) accelerated simulation was performed and MD trajectories were subjected to post-simulation analysis by using VMD and Chimera [53,54].

### 3.6. In Silico ADMET Analysis

The physiochemical, pharmacokinetics, toxicity, and cross-reactivities of five selected compounds were predicted by ADMETlab 2.0 [55]. The SMILES notation of all the compounds was subjected to the submission webpage of ADMETlab for the calculation of the above-mentioned properties.

## 4. Conclusions

Herein, we employed a multistep virtual screening strategy to screen an in-house database of biologically active natural and synthetic compounds in the pursuit of potent Omicron Spike protein inhibitors. In the result of docking-based screening, five compounds were identified with significant binding affinity and concurrently occupy the receptor-binding domain of Omicron’s Spike protein. Binding mode analysis of selected hit compounds demonstrated the different binding modes; however, the interacting crucial residues were similar. Moreover, the DFT calculations reveal the molecular properties and chemical reactivity of the selected compounds. Further, MD simulation studies indicated the conformational stability of the RBD domain of Omicron’s Spike protein upon binding of selected virtual hits. These findings could be utilized to further in vitro and in vivo studies to validate the potencies of these compounds against SARS-CoV-2 variants.

## Figures and Tables

**Table 1:** Two-dimensional structures, docking scores and comprehensive interactions between RBD of Spike protein and the selected hit compounds.

Name	Structures	Dock Score (kcal/mol)	Hydrogen Bonding Interactions				Van der Waals Interactions
			Donor	Donor H	Acceptor	Distance (Å)	
**AKS-01**	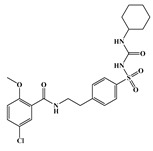	**−6.94**	Arg403:NH1 Arg403:NH2 Arg408:NH2 Lig:N1 Arg493:NH2 Ser496:H Tyr501:OH Lig:N1 Lig:N2	Arg403:H11 Arg403:H21 Arg408:H21 Lig:H3 Arg493:H22 Ser496:H22 Tyr501:H Lig:H12 Lig:H13	Lig:O4 Lig:O4 Lig:O Asn417:OD1 Lig:O1 Lig:O2 Lig:N2 His505:ND1 His505:ND1	2.1 2.6 3.4 3.7 3.8 2.6 3.1 2.4 2.2	Arg403, Asn417, Tyr453, Tyr495
AKS-02	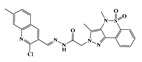	−6.46	Arg493:NE Arg493:NH2 Ser496:N Gly502:N Lig:N3	Arg493:HE Arg493:H22 Ser496:H Gly502:H Lig:H9	Lig:O Lig:O Lig:O1 Lig:N5 His505:ND1	3.1 3.2 3.3 2.1 3.1	Tyr453, Tyr495, Tyr501
AKS-03	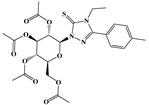	−6.03	Tyr501:OH	Tyr501:H	Lig:O4	2.5	Arg403, Arg493, Tyr453, Tyr495, Tyr501, His505
AKS-04	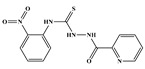	−5.47	Arg493:NH1 Arg493:NH2 Lig:N1 Lig:N3	Arg493:H11 Arg493:H21 Lig:H4 Lig:H6	Lig:O Lig:O Ser496:OG His505:ND1	2.9 3.1 3.0 2.1	Arg403, Tyr449, Tyr450, Tyr495
AKS-05	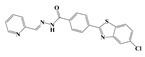	−5.24	Gly502:N Lig:N1	Gly502:H Lig:H7	Lig:N3 His505:ND1	2.1 2.9	Arg403, Asn417, Tyr453, Leu455, Tyr501, His505

**Table 2:** Thermodynamics parameters and molecular orbital energy values of selected hit compounds.

Name	HOMO (eV)	LUMO (eV)	Band Gap (eV)	Diploe Moment	Total Energy (eV)
AKS-01	−6.911	−1.442	5.442	4.95	−62,638.5
AKS-02	−6.312	−2.367	3.918	3.59	−63,688.1
AKS-03	−5.823	−1.469	4.326	8.73	−60,226.9
AKS-04	−6.585	−3.210	3.346	5.52	−38,090.5
AKS-05	−6.639	−2.639	3.972	4.37	−52,322.4

**Table 3:** In silico predicted physiochemical, pharmacokinetic, toxicity, and cross-reactivities of the five selected virtual hits.

Compound	Mol. Weight g/mol	nHA	nHD	TPSA	LogP	Lipinski Rule	BBB Penetration	Pg Substrate	Acute Toxicity Rule	AMES Toxicity	HIA	PAINS
AKS-01	493.140	8	3	113.6	3.824	Accepted	Yes	No	0 alerts	Non toxic	Yes	0 alerts
AKS-02	508.110	9	1	109.5	4.424	Accepted	Yes	Yes	0 alerts	Non toxic	Yes	0 alerts
AKS-03	549.180	12	0	137.1	2.523	2 alerts	Yes	Yes	0 alerts	Non toxic	Yes	0 alerts
AKS-04	317.060	8	3	109.1	1.625	Accepted	No	Yes	0 alerts	Toxic	Yes	0 alerts
AKS-05	392.050	5	1	67.24	4.546	Accepted	Yes	Yes	0 alerts	Toxic	Yes	0 alerts

## Data Availability

Not applicable.
